# Sequence Analysis of Egyptian Foot-and-Mouth Disease Virus Field and Vaccine Strains: Intertypic Recombination and Evidence for Accidental Release of Virulent Virus

**DOI:** 10.3390/v12090990

**Published:** 2020-09-06

**Authors:** Sahar Abd El Rahman, Bernd Hoffmann, Reham Karam, Mohamed El-Beskawy, Mohammed F. Hamed, Leonie F. Forth, Dirk Höper, Michael Eschbaumer

**Affiliations:** 1 Department of Virology, Faculty of Veterinary Medicine, Mansoura University, Mansoura 35516, Egypt; sahar_virol@mans.edu.eg (S.A.E.R.); rehamviro@gmail.com (R.K.); 2 Institute of Diagnostic Virology, Friedrich-Loeffler-Institut, 17493 Greifswald-Insel Riems, Germany; bernd.hoffmann@fli.de (B.H.); leonie.forth@tobka.de (L.F.F.); dirk.hoeper@fli.de (D.H.); 3 Department of Animal Medicine, Faculty of Veterinary Medicine, Matrouh University, Matrouh 51744, Egypt; melbeskawy@gmail.com; 4 Department of Pathology, Faculty of Veterinary Medicine, Mansoura University, Mansoura 35516, Egypt; mohamedpathology@gmail.com

**Keywords:** FMDV, Egypt, full-genome sequencing, intertypic recombination, vaccine composition analysis, frozen evolution

## Abstract

In spite of annual mass vaccination programs with polyvalent inactivated vaccines, the incidence and economic impact of foot-and-mouth disease (FMD) in Egypt is high. Viruses of the A, O and SAT 2 serotypes are endemic and repeated incursions of new lineages from other countries lead to an unstable situation that makes the selection of appropriate vaccine antigens very difficult. In this study, whole genome sequencing of a 2016 serotype A isolate from Egypt revealed a recombination event with an African serotype O virus. Based on available vaccine matching data, none of the vaccines currently used in Egypt are expected to sufficiently protect against this virus or other viruses of this lineage (A/AFRICA/G-IV) circulating there since 2012. In addition to the risk of vaccine failure caused by strain mismatch, the production of inactivated FMD vaccines is dangerous if adequate biosafety cannot be maintained. Using a high-throughput sequencing protocol optimized for short nucleic acid fragments, the composition of a local inactivated vaccine was analyzed in depth. The serotype O strain identified in the vaccine was genetically identical to viruses found in recent FMD outbreaks in Egypt.

## 1. Introduction

Foot-and-mouth disease (FMD), caused by a highly contagious viral pathogen that belongs to the genus *Aphthovirus* in the family *Picornaviridae* [1], is a major burden of agriculture and trade worldwide [2]. It affects cloven-hoofed livestock, in which fever, painful vesicular lesions, lameness, milk drop and loss of condition lead to major losses in production.

FMD virus (FMDV) particles have a small icosahedral capsid formed by four structural proteins that enclose an RNA genome of 8.4 kb in size. The genome encodes a polyprotein that is co- and post-translationally processed into the structural (VP1-4) and nonstructural (leader, 2A-C, 3A-D) proteins [1,3].

FMDV spreads predominantly by direct or indirect contact with infected animals and their secretions or by contaminated feed. Under certain circumstances, airborne dissemination of infectious aerosols can occur over long distances [4]. The main tool for FMD control especially in enzootic localities is vaccination. Current vaccines are chemically inactivated but structurally intact virions [5] selected to match the viruses circulating in a specific area or region [2]. This is challenging not only because there are seven immunologically distinct serotypes of FMDV: O, A, C, Asia 1 and Southern African Territories (SAT) 1, SAT 2 and SAT 3. Like many RNA viruses, FMDV has high genetic variability, caused by error-prone RNA replication and extensive intra- and inter-serotype recombination [1,6]. Within each serotype, the many subtypes and lineages are evolving rapidly and are spread within and between known epidemiological clusters (so-called virus pools) by trade in live animals and animal products [1]. Serotype C may be extinct in the wild, SAT 3 is mostly restricted to Southern Africa and serotype Asia-1 has only been found on that continent, but the serotypes O, A, SAT 1 and SAT 2 are widespread in Africa [2]. FMDV is endemic in Egypt and neighboring countries, and viruses of serotypes O, A and SAT 2 have been detected in domestic and wild animals in the last decade [7]. In this study, recent Egyptian virus isolates and locally produced vaccines were analyzed to better understand the epidemiological situation in the region and support effective FMD control measures.

## 2. Materials and Methods

### 2.1. Field Samples and Inactivated Vaccine

A panel of 62 samples (whole blood, serum, buffy coat, nasal and saliva or oral swabs) was collected from ruminants in the Delta region of Egypt (Dakahlia and Damietta governorates) in 2016. This included saliva samples from two cattle (*Bos taurus*) and two river buffalo (*Bubalus bubalis*) with clinical signs suspected to be FMD.

In 2019, two domestically produced trivalent inactivated FMD vaccines (from different manufacturers) were procured for the composition analysis. The vaccines were spotted (200 µL per spot) on FTA cards (Sigma-Aldrich, Munich, Germany), air-dried and shipped from Egypt to Germany, where a quarter of each spot was used for RNA extraction.

### 2.2. FTA Card Storage Experiment

For comparison, another commercial inactivated FMD vaccine (produced in the European Union for the global market; previously used in the study by Forth et al. [8]) was spotted on two FTA cards (one spot per card, 125 µL per spot) in the laboratory in Germany, air-dried and stored at room temperature. A quarter of each spot was excised after 2 h, 4 weeks, 2 months and 6 months, respectively, and used for RNA extraction. The recovery of FMDV RNA from the card was calculated from the mean C_q_ values of the original material and the RNA extracted from the excised spots by the following formula:

(1) $$\%\mathrm{recovery}={1/2}^{\mathrm{mean}\left(\mathrm{Cq}\;\mathrm{FTA}\;\mathrm{card}\right)-\left(\mathrm{mean}\left(\mathrm{Cq}\;\mathrm{liquid}\;\mathrm{vaccine}\right)+2\right)}\times 100$$

This takes into account that one volume of the original vaccine was applied to the card, but only a quarter of the spot was processed at each time point.

### 2.3. RNA Extraction

#### 2.3.1. Liquid Samples

RNA was extracted from 100 μL of each animal sample with the NucleoMag VET kit (Macherey-Nagel, Düren, Germany) on a KingFisher Flex magnetic particle processor (Thermo Fisher Scientific, Waltham, MA, USA).

#### 2.3.2. FTA Cards

For RNA extraction from FTA cards, the excised paper was cut into smaller fragments, suspended in 1 mL of TRIzol Reagent (Thermo Fisher Scientific, Waltham, MA, USA) and ground with a 5-mm steel ball in a TissueLyser bead mill (Qiagen, Hilden, Germany). Chloroform was added to the tube, the contents were thoroughly mixed and then centrifuged. DNA-free total RNA was extracted from the supernatant using RNeasy Mini spin columns (Qiagen) with on-column DNase I digestion as recommended by the manufacturer.

### 2.4. FMDV Real-Time RT-PCR

To quantify viral RNA content in animal samples and FTA cards, extracted RNA was used for real-time RT-PCR (targeting a conserved locus in the 3D-coding region of the genome) [9] as previously described [10].

### 2.5. Virus Isolation

Animal samples that were positive in the FMDV real-time RT-PCR were used for virus isolation as previously described [10]. Briefly, confluent monolayer cultures of baby hamster kidney cells (BHK-21) and highly sensitive recombinant porcine kidney cells expressing bovine αvβ6 integrin (LFBK-αvβ6; [11]) were inoculated with 100 µL of sample and incubated for 3 days. The cultures were then frozen and thawed and 200 µL of the lysate were used to inoculate a second set of confluent BHK-21 and LFBK-αvβ6 cultures. The manifest cytopathic effect in the passage was considered indicative of viral replication; this was confirmed by RNA extraction and FMDV real-time RT-PCR as described above.

### 2.6. Viral Genome Sequencing

#### 2.6.1. VP1-Coding Region

The nucleotide sequence of the 1D region of the FMDV genome, which codes for the capsid protein VP1 [3], was obtained using the primers FMD-3161-F and FMD-4303-R previously described by Dill et al. [12].

#### 2.6.2. Full-Length Viral Genome

One of the virus isolates (from sample 10, 1st passage on LFBK-αvβ6 cells) was used for whole genome sequencing. Total RNA was extracted from the culture supernatant and used for sequence-independent single-primer amplification by a previously described method [13] with small modifications. First, cDNA was prepared with the qScript Flex cDNA Synthesis Kit (Quantabio, Beverly, MA, USA) and the K-8N primer (5′ GACCATCTAGCGACCTCCACNNNNNNNN 3′), from which dsDNA was synthesized with the same primer and the Klenow fragment of *E. coli* DNA Polymerase I (New England Biolabs, Ipswich, MA, USA). The dsDNA was purified with sparQ PureMag beads (Quantabio) and used as the template for PCR amplification with Phusion High-Fidelity polymerase and the K primer (5′ GACCATCTAGCGACCTCCAC 3′). The PCR product was cleaned up with a QIAEX II column (Qiagen), quantified with the QuantiFluor dsDNA system (Promega, Madison, WI, USA) and submitted to Eurofins, Constance, Germany for Ilumina paired-end sequencing.

#### 2.6.3. Vaccine Composition Analysis

A DNA library was prepared from the RNA extracted from one of the FTA cards from Egypt and sequenced as previously described [8]. Briefly, the RNA quality was assessed using an Agilent 2100 Bioanalyzer, and approximately 200 ng of RNA concentrated with Agencourt RNAClean XP beads (Beckman Coulter, Brea, CA, USA) were used for cDNA synthesis with the SuperScript IV First-Strand cDNA Synthesis System (Thermo Fisher Scientific) and the NEB Next Ultra II Non-Directional RNA Second-Strand Synthesis Module (New England Biolabs). The cDNA was fragmented using a Covaris M220 Focused-ultrasonicator (Covaris, Woburn, MA, USA) with a target size of 400 bp. Sheared DNA was concentrated with Agencourt AMPure XP beads (Beckman Coulter) and used for library preparation with the GeneRead DNA Library L Core kit (Qiagen). After end repair, adapter ligation, size selection and quality control, small- and large-fragment libraries were sequenced in pooled runs on an IonTorrent S5 XL instrument with an Ion 530 Chip Kit (Thermo Fisher Scientific).

Both datasets were combined for a de novo assembly using GS De Novo Assembler (454 Life Sciences/Roche, Penzberg, Germany) as described [8]. The correct assembly was verified by stringent mapping to the assembled sequence with the GS Reference Mapper (454 Life Sciences/Roche). In addition, the metagenomic software pipeline RIEMS [14] was applied to the combined dataset. Reads identified as FMDV by megablast were sorted and counted according to their best hit. Based on the megablast results, two reference sequences (accession# KP940473 and KC440884) were selected and used for reference guided-assembly with the GS Reference Mapper.

#### 2.6.4. Nucleotide Sequence Alignments and Phylogenetic Analysis

All nucleotide sequence alignments in this study were performed with MUSCLE [15] 3.8.425 in Geneious Prime 2019.2.3 (https://www.geneious.com/home/). Approximately maximum-likelihood phylogenetic trees were inferred from the alignments with FastTree [16] 2.1.11 using the generalized time-reversible model of nucleotide evolution. All full-length nucleotide sequences from four FMDV serotypes (O, A, Asia 1 and SAT 2) that were available from the GenBank of the U.S. National Institutes of Health (NIH; https://www.ncbi.nlm.nih.gov/genbank/) were used for whole genome analyses. For VP1 phylogenies, a subset of available 1D nucleotide sequences was selected to put the newly described virus isolates into a global context while also giving adequate representation to contemporary and historical isolates from the North and East African regions. Topotypes and lineages were annotated following the guidance of the World Reference Laboratory (WRL) for FMD at The Pirbright Institute, United Kingdom (https://www.wrlfmd.org/fmdv-genome/fmd-prototype-strains).

An explorative recombination analysis using an alignment of four full-length FMDV genomes—sample 10 from this study, A/El-Fayoum/Egypt/2014 (accession# KP940474), O/AMU_200/Uganda/2017 (MH791293) and SAT2/EGY/24/2014 (KY825720)—was performed using RDP4 [17]. The alignment was cropped to the shortest sequence (KY825720) and then contained 7229 nucleotide (nt) positions, from 169 nt upstream of the leader-coding region to the stop codon at the 3′ end of the 3D-coding region. This was used for a distance plot, where a window of 200 nt in length was moved by steps of 20 nt along the alignment. Pairwise distances were calculated for each window using DNADIST (a component of the PHYLIP package in RDP4) and then plotted against the alignment position of the center of the window.

## 3. Results and Discussion

### 3.1. Animal Samples

The saliva samples from the two cattle and two buffalo suspected to have FMD (sample nos. 8, 9, 10 and 11; see Table 1) were positive in the FMDV real-time RT-PCR with C_q_ values of 28.2, 38.0, 22.0 and 37.1, respectively. The remaining 58 animal samples were negative in the PCR.

Of the PCR-positives, the most strongly positive sample (no. 10) was also positive in the virus isolation in both cell lines. Samples 8 and 11 were only positive in LFBK-αvβ6, but not in BHK-21 cells, and sample 9 was negative in both.

Complete 1D nucleotide sequences (coding for the capsid protein VP1) were obtained from the original RNA of all four samples as well as from all virus isolates (1st and 2nd passage). The original sequences were deposited in the NIH GenBank (see Table 1 for accession numbers).

The sequences of samples 8 and 9 from the original RNA were identical. Due to the low virus content, no isolate was obtained for sample 9, but the sequence of the virus isolated from sample 8 did not change over two passages in LFBK-αvβ6 cells. The nucleotide sequences of samples 10 and 11 were identical in the original RNA and distinct from samples 8 and 9, with an adenine instead of a cytosine at position 372 in 1D. The VP1 amino acid sequences of all four samples, however, were the same because both bases at this position code for an arginine at residue 124.

The consensus 1D sequence of sample 10 was stable over two passages in BHK-21 and LFBK-αvβ6 cells; therefore, this isolate was selected for whole genome analysis (see below). The sequence of sample 11 did change upon isolation in LFBK-αvβ6 cells, with an adenine (A) replacing a guanine (G) at position 25 in 1D, resulting in a substitution of the aspartate (D) residue at position 9 of VP1 with an asparagine (N). This exchange was maintained over an additional passage in LFBK-αvβ6 cells. Amino acid exchanges in the capsid proteins during cell culture adaptation of field viruses are common, but this is the first description of an exchange at position 9 of VP1 caused by cell culture adaptation of FMDV, and the first report of an N residue at this position [18].

**Figure 1:**
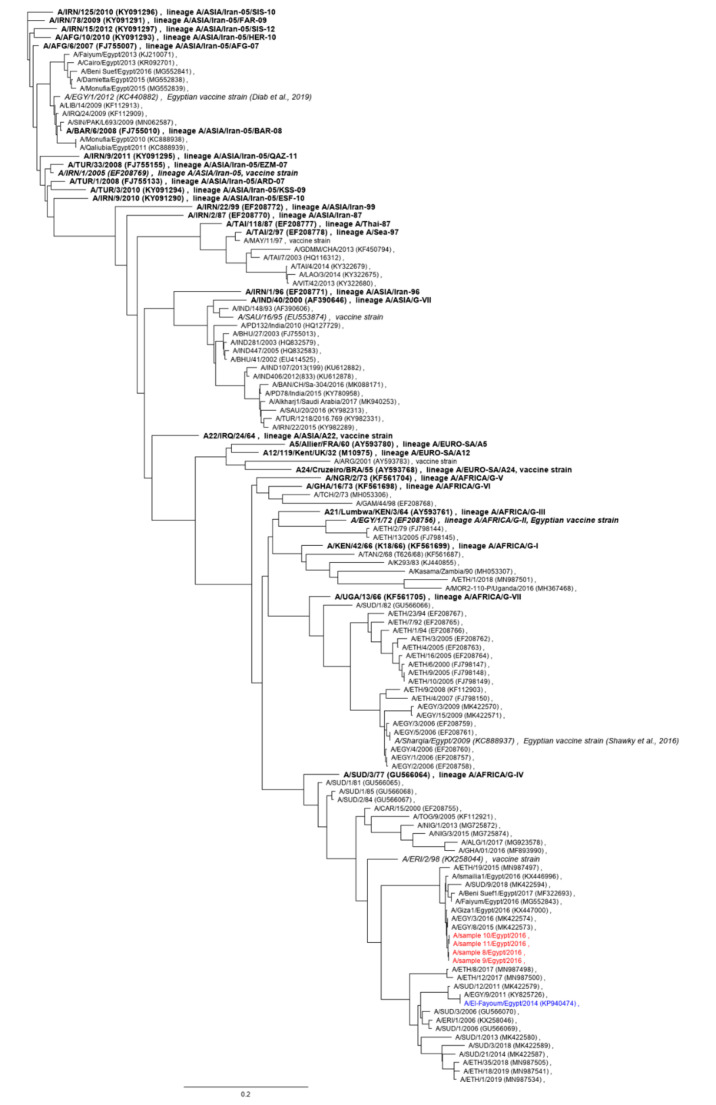
Serotype A 1D (VP1) phylogeny. Newly described viruses from this study are shown in red, the defining historical isolates for each topotype or lineage of FMDV A are in bold print and relevant vaccine strains in italics. A/El-Fayoum/Egypt/2014 is shown in blue.

Based on their VP1-coding sequences, the 2016 serotype A FMDV isolates from the Delta region of Egypt from this study are closely related to previously described Egyptian viruses from this time period (Figure 1), with 99.8% nucleotide sequence identity and 99.0% shared amino acids with FMDV A/Giza 1/EGY/2016 (accession# KX44700). Based on a genotyping report of the World Reference Laboratory (WRL) for FMD [19], these viruses belong to the lineage A/AFRICA/G-IV and other closely related isolates include FMDV A/EGY/3/2016 and A/EGY/19/2016. For the latter isolates, vaccine matching data are available from the WRL [20,21], indicating that the A/ASIA/Iran-05 lineage strains that are widely used as vaccines in Egypt (e.g., A/EGY/1/2012) [22,23] will not provide useful protection against infection with these viruses. Similarly, available in vitro matching data suggest that A Saudi-95 is ineffective against recent strains of the A/AFRICA/G-IV lineage from Egypt, Sudan and Ethiopia [24]. It is important to note that the vaccine match cannot be accurately predicted using the number of amino acid substitutions alone [25], but the considerable genetic distance between sample 10 and A/EGY/1/2012 (76% shared nucleotides in 1D, 85% identical amino acids in VP1) or sample 10 and A/SAU/16/95 (79%/87%) does suggest appreciable antigenic distance. In addition to in vitro tests, in vivo challenge studies should be conducted to assess the efficacy of the available vaccines against circulating strains. It has been previously shown that high-potency vaccines against FMDV serotype A can induce in vivo protection against heterologous strains despite poor in vitro matching results [26], but there are no publicly available data on the potency of the vaccines used in Egypt. There are published vaccination and challenge studies (e.g., [27] or [22]), but the authors are not aware of any that followed the guidelines for FMD vaccine efficacy estimation [28] of the World Organisation for Animal Health.

The local vaccine producer MEVAC advertises three polyvalent inactivated FMDV vaccines that include an “A Africa” strain [23]. The identity of this strain, or even its lineage, is unknown. While it could be a recent isolate of lineage A/AFRICA/G-IV, another origin is more likely: The Veterinary Serum and Vaccine Research Institute (VSVRI) in Cairo, which also supplies vaccines for the official FMD vaccination campaigns in Egypt, offers a bivalent formulation containing “O1/93“ and “AEGY/06” [29]. The former is O_1_ EGY/3/93 (accession# EU553840) of lineage O/ME-SA and the latter is one of the 2006 isolates of lineage A/AFRICA/G-VII described by Knowles et al. [30], alternatively referred to as A/Sharqia/Egypt/2009 [31]. (A full-length sequence is available under accession# JF749843.) These viruses are only distantly related to the 2016 isolates from this study (79% nucleotide and 85% amino acid identity between sample 10 and A/Sharqia/Egypt/2009), again suggesting little or no cross-protection, but no data on the antigenic relatedness between the G-VII vaccine and the G-IV field strains are available.

In summary, it appears likely that none of the serotype A FMD vaccines currently marketed and used in Egypt are actually effective against the viruses of lineage A/AFRICA/G-IV recently circulating in the country. There is a commercial A/AFRICA/G-IV vaccine strain (A/ERI/2/98), which is more closely related genetically (87% identity in 1D and 91% in VP1) and gave promising in vitro matching results against contemporary isolates of this lineage from North Africa [32], but it is not known to the authors whether this strain is available for use in Egypt.

### 3.2. Full-Length Sequence of Serotype A Isolate from 2016

An 8196 nt full-length sequence (including a 20 nt poly-C tract [3] and a 147 nt poly-A tail) of the virus isolated from sample 10 (1st passage on LFBK-αvβ6 cells) was obtained by deep sequencing and submitted to GenBank (accession# MT863268, Table 1).

**Figure 2:**
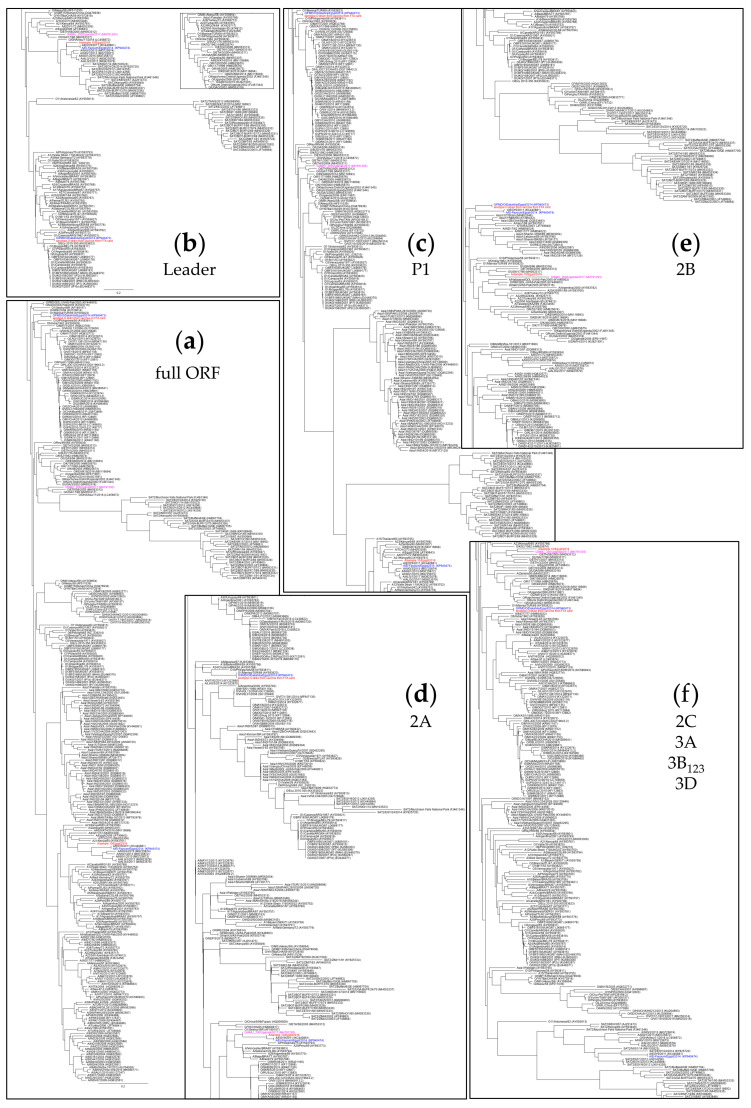
Phylogenetic trees for (**a**) the full open reading frame (ORF), (**b**) the leader protein-coding region, (**c**) the P1 region coding for the capsid proteins, as well as for the genomic regions coding for (**d**) 2A, (**e**) 2B and (**f**) 2C to 3D of the viruses in this study. In each panel, sample 10 and the serotype O strain from the inactivated vaccine are highlighted in red and their closest relatives for which full-length sequences are available (A/El-Fayoum/Egypt/2014, accession# KP940474 and O/Dakhalia/Egypt/2014, accession# KP940473; [33]) are marked in blue. O/AMU_200/Uganda/2017 (accession# MH791293) is shown in purple. The trees have been cropped to the area of interest; uncropped trees are available as Figures S1–S6. A small section of the tree for the leader-coding region that includes O_1_/Manisa/TUR/69 is shown as an insert in panel (b); see Figure S2 for the full tree.

Phylogenetic trees for the entire polyprotein open reading frame (ORF), the leader protein-coding region, the P1 region coding for the capsid proteins, as well as the 2A, 2B and 2C to 3D coding regions of FMDV O, A, Asia 1 and SAT 2 are shown in Figure 2. Among the close relatives of the 2016 serotype A samples seen in the 1D (VP1) phylogenetic tree in Figure 1, the closest one where the full sequence of the ORF is publicly available is A/El-Fayoum/Egypt/2014 (accession# KP940474; [33]) (Figure 2a). This isolate is marked in blue in the phylogenetic trees in Figure 2, and the sample 10 sequence is marked in red. Across the entire ORF, the two viruses shared 87.3% of nucleotides (Table 2).

In the leader protein-coding region, the isolate A/El-Fayoum/2014 and sample 10 from this study were closely related (Figure 2b). They also clustered closely with serotype O strains from Africa as well as East and North African isolates of SAT 2. The SAT 2 isolates from Southern Africa, however, were quite distinct.

In the P1 (capsid protein) coding region, A/El-Fayoum/2014 and sample 10 were also very closely related, as was already seen in the 1D (VP1) phylogeny in Figure 1. The P1 coding sequences clustered strictly by serotype, so the A viruses were far removed from either O, SAT 2 or Asia 1 (Figure 2c). The phylogenies of the individual capsid protein coding regions were very similar to that of the entire P1 region (not shown). Structural constraints of the FMDV capsid probably prevent recombination breakpoints within P1 [6,34], but it is bounded by two breakpoint hot spots. It has been proposed that P1 is a functionally interchangeable module that undergoes promiscuous recombinatorial exchange [35].

**Figure 3:**
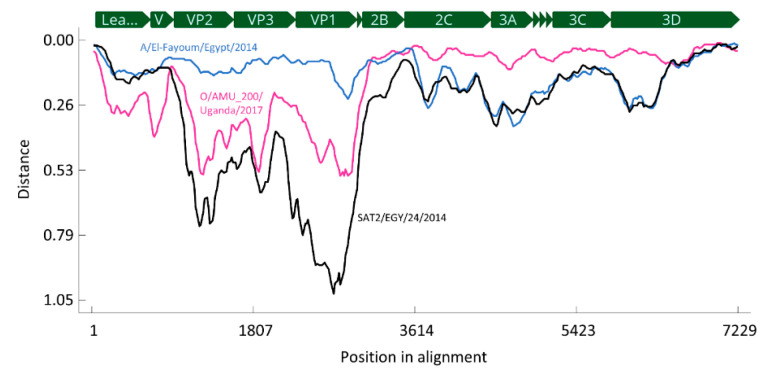
Distance plot of the serotype A virus from sample 10 against A/El-Fayoum/Egypt/2014 (blue), O/AMU_200/Uganda/2017 (purple) and SAT2/EGY/24/2014 (black).

The 2A-coding region is very small and there were several distinct clusters with high conservation within each cluster, which did not correlate with the serotype (Figure 2d). In this genomic region, A/El-Fayoum/2014 and sample 10 were very closely related, and their nearest relative was a serotype O isolate from Uganda from 2017 (O/AMU_200/Uganda/2017, accession# MH791293, [36]; marked in purple in the figure). This serotype O virus was also the closest known relative to sample 10 in the remainder of the genome downstream of the 2A-coding region (Figure 2e,f and Figure 3; Table 2), in marked contrast to A/El-Fayoum/2014.

In the 2B-coding region, A/El-Fayoum/2014 actually was closest to O/Dakhalia/Egypt/2014 (Figure 2e), whereas in the large region coding for the non-structural proteins 2C to 3D, it was most closely related to SAT2/EGY/24/2014 (accession# KY825720; Figure 3) and other SAT 2 isolates from Egypt and Palestine (Figure 2f and Figure 3).

These observations point towards frequent recombination between FMD viruses of different serotypes cocirculating in endemic regions in Africa. It is important to remember that recombination requires that an animal (indeed the same cell) must be infected with two strains of FMDV at the same time. It is presently unclear whether recombination only occurs when two viruses cause an acute infection at the same time or if the superinfection of a persistently FMDV-infected carrier animal with a different virus strain will lead to the same outcome [6].

**Figure 4:**
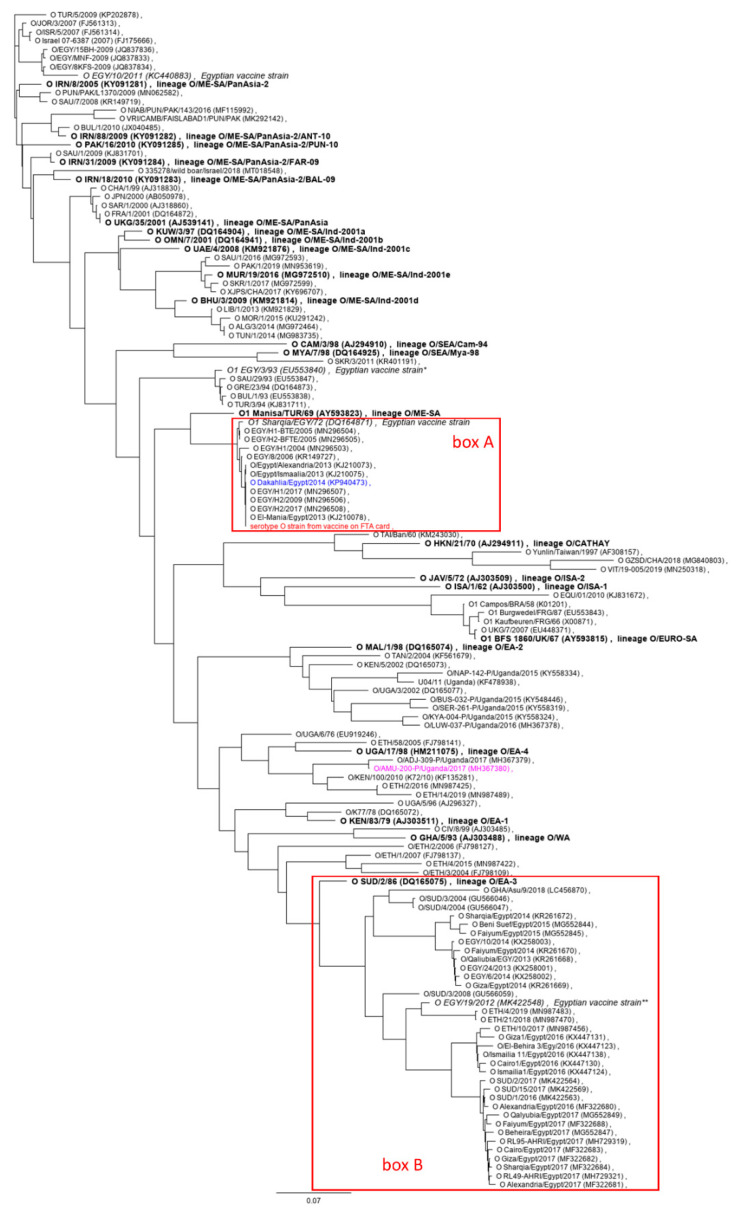
Serotype O 1D (VP1) phylogeny. The serotype O vaccine strain recovered from the FTA card is shown in red, the defining isolates for each topotype or lineage of type O are in bold print and known or suspected vaccine strains used in Egypt are in italics. O/AMU_200/Uganda/2017 is shown in purple and O/Dakhalia/Egypt/2014 is shown in blue. * The O/EGY/3/93 sequence from accession# EU553840 was used to represent the closely related O EGY 2/93 vaccine strain [41,42] instead of the shorter homologous sequence fragment (accession# AJ303468). ** The actual virus isolate used for the O/EA-3 vaccine described by Shafik et al. [22] is O/EGY/4/2012, whose VP1 sequence is not publicly available. In its stead, O/EGY/19/2012 was used for the phylogenetic analysis because it is the only 2012 O/EA-3 isolate from Egypt in the GenBank.

Due to the scarcity of publicly available full-genome sequence data of African FMDV isolates, it is impossible to determine when and where this recombination has occurred. It would certainly be a misinterpretation of the data to suggest that the virus from sample 10 (isolated from a river buffalo in Damietta Governorate, Egypt in 2016) and O/AMU_200/Uganda/2017 were immediately involved in this event. To the contrary, the considerable genetic distance between sample 10 and its closest relatives in the database (Table 2) indicates that there is a large gap in the publicly available sequence data and that we are missing information on many intermediate viruses. There have been no reports yet of O/EA-4 viruses like O/AMU_200 circulating in Egypt, but it is worth pointing out that closely related viruses of this lineage have been found in Ethiopia (Figure 4).

Recombination between FMDV field strains of different serotypes has been documented previously [6,37,38,39], but this is the first report of an intertypic recombinant from Egypt. As has been pointed out by other authors, it is very important to recognize these events to avoid biases in phylogenetic analyses and vaccine selection [34,38,40]. The VP1-coding region only comprises of 8% of the FMDV genome and if analyses are restricted to this region, a large proportion of the genetic information is not accounted for [34].

### 3.3. FTA Card Storage Experiment

In the FTA card storage experiment, the original vaccine preparation had a mean FMDV C_q_ value of 15. When the paper was processed 2 h after the vaccine had been applied to the card, about 15% of the RNA could be recovered (mean C_q_ value 19.7). After 4 weeks, only about 2% of the original FMDV RNA was recovered from the card and this did not further decrease during another month (mean C_q_ value 22.6 and 22.4, respectively). By six months after application of the vaccine to the FTA card, recovery had decreased to 1% (mean C_q_ value 23.8).

### 3.4. Composition Analysis of 2019 Inactivated FMDV Vaccine

The Egyptian vaccines spotted on the FTA cards had C_q_ values in the FMDV real-time RT-PCR of 34.6 and 28.3. Assuming that the decrease in recoverable FMDV RNA was the same between the FTA cards prepared in Egypt and the storage experiment in the laboratory, the estimated C_q_ values of the original vaccine preparations were 27 and 21, a considerably lower FMDV RNA content than in the vaccine used by Forth et al. [8], which had a C_q_ of 15. For a standard inactivated and purified vaccine, FMDV RNA content and antigen payload should be highly correlated (one genome copy per virus particle), suggesting a much lower payload in the Egyptian vaccines.

Only the RNA with the lower C_q_ value was used for the composition analysis. An 7787 nt sequence of a serotype O strain (without the S-fragment [3], poly-C tract or the poly-A tail) was reconstructed from 46,057 FMDV reads in the library and submitted to GenBank (accession# MT863269, Table 1). In addition, 37 reads mapping to FMDV SAT 2 (best match SAT2/EGY/3/2012, accession# KC440884) were identified in the sample. This mapping resulted in eight contigs (in total 1509 bases) covering approximately 21% of the SAT 2 reference sequence. No evidence of any other serotype or other isolates of serotypes O or SAT 2 was detected. This is at odds with the manufacturer’s description of the vaccine, which was advertised as a trivalent preparation containing FMDV serotypes A, O and SAT 2. It is undetermined why serotype A was not detected in the vaccine sample on the FTA card. In previous experiments, minor components with less than 7% representation in the vaccine formulation had been successfully detected by the composition analysis [8], but the FMDV RNA content of the sample was much higher than in the present study. The metagenomic analysis also revealed a considerable amount of bacterial contamination (70% of reads), but it cannot be excluded that this occurred during or after application of the vaccine to the FTA card and was not present in the original vaccine.

Poor sample quality can reduce the sensitivity of the composition analysis and is a possible explanation for the small number of identified SAT 2 reads and the apparent absence of serotype A from the preparation. Nevertheless, since the degradation of FMDV nucleic acid in the sample was not serotype-specific, it can be safely concluded that the original preparation indeed contained a much higher amount of serotype O RNA than any other serotype. In turn, the high abundance of serotype O reads allowed the reconstruction of a reliable consensus sequence, which is unlikely to be affected by the low sample quality because random errors in individual reads are compensated by other overlapping reads.

Based on its VP1-coding sequence (Figure 4), the virus from the inactivated vaccine (of lineage O/ME-SA, box A) was very distant from the lineage O/EA-3 strains (box B) now circulating in Egypt and elsewhere in North Africa. An isolate that is closely related to the vaccine strain from this study, O_1_ Sharqia/EGY/72, has been used as a vaccine strain in Egypt for decades [42]. No vaccine matching data for this strain against contemporary O/EA-3 viruses are publicly available, but good in vitro matches have been reported for the related O_1_ Manisa strain [24]. A homologous O/EA-3 vaccine containing the O/EGY/4/2012 isolate (Figure 3) has been produced by VSVRI [22], but it is unknown to the authors whether this is still being offered. One product containing O/EA-3 is available from MEVAC [23].

MEVAC also offers several products that contain O/PanAsia-2 [23], but as with their “A Africa” vaccine discussed above, the identity of this strain has not been publicly disclosed. VSVRI refers to its cognate strain as being from the “O PanAsia outbreak 2011” [43]. There is no record of an O/PanAsia outbreak in Egypt in 2011. However, O/PanAsia-2 was reported that year [2,44], so this may be a clerical error and the VSVRI vaccine strain probably is O/EGY/10/2011 (accession# KC440883) or a close relative. The confusion is compounded by the fact that elsewhere on the linked page [43] the strain in the vaccine is referred to as subtype “O1”, which would match, e.g., O_1_/EGY/93 or O_1_/Sharquia/EGY/72 (and presumably the vaccine virus identified in this study), but these do not belong to either the PanAsia or PanAsia-2 sublineages of the O/ME-SA topotype [45].

Similar to what has been described above for the serotype A isolate from sample 10, the phylogenetic context of the serotype O strain from the inactivated vaccine was variable across the genome (see Figure 2). While it was generally closely related to other O/ME-SA isolates (e.g., the O_1_ Manisa or O_1_/Sharqia/EGY/72 vaccine strains), this was not at all the case in the genomic regions coding for the leader and 2B nonstructural proteins. The leader protein-coding region of the vaccine strain from this study was closely related to other historical EURO-SA vaccine strains such as O_1_/Campos/BRA/58 or O_1_/BFS1860/UKG/67, but not to O_1_/Manisa/TUR/69, which clusters with historical ME-SA strains such as O_5_ India or O_10_ Philippines (Figure 2b and Figure S2). As mentioned above, in the 2B-coding region, the serotype O vaccine strain from this study (like O/Dakhalia/Egypt/2014) was actually closest to the A/El-Fayoum/Egypt/2014 isolate described by Sobhy et al. [33] (Figure 2e).

The serotype O vaccine strain from this study is a reasonably close match (98.9% nucleotide identity, five amino acid exchanges in VP1) with the known Egyptian vaccine strain O_1_ Sharqia/EGY/72 (accession# DQ164871). At the same time, it was virtually identical to a field strain from 2014 (O/Dakhalia/Egypt/2014, accession# KP940473, [33]; see also Figure 2a). Across the entire ORF, there was only one point mutation (A → G) leading to one amino acid exchange at residue 91 of 3A (N → serine, S). This strain was isolated from sloughed bovine tongue epithelium from a clinical case in the Dakhalia governorate in winter 2014/2015 [33]. No other full-length sequence of a closely related field isolate was publicly available, but there were several 100% identical 1D sequences in the NIH database, both from earlier (e.g., O/EGY/H2/2009, accession# MN296506 or O/El-Mania/Egypt/2013 and accession# KJ210078) and later outbreaks (O/EGY/H1/2017, accession# MN296507 and O/EGY/H2/2017 and accession# MN296508; box A in Figure 3). These were also very closely related to O/EGY/8/2006 (KR149727; 99.7% nucleotide identity in 1D/VP1, one silent mutation and one amino acid exchange). The clinical isolates O/EGY/H1-BTE/2005 (accession# MN296504) and O/EGY/H2-BFTE/2005 (accession# MN296504) were again identical and even more similar (one amino acid exchange in VP1) to the much older O_1_/Sharquia/EGY/72 isolate, as has been previously noted by Abu-Elnaga et al. [46]. While this may be a result of widespread laboratory contamination and misidentification of isolates, it appears unlikely considering the diversity of researchers and institutions that have contributed to the database.

This high degree of sequence conservation over decades is hard to reconcile with the well-established high evolutionary rate of FMDV [1] (about 0.5–1% of the genome per year [6]). In fact, such unnaturally close relationships between vaccine strains and field isolates of FMDV have been identified before and were found to be caused by either the accidental release of FMDV from a laboratory or vaccine production facility or by inadequate inactivation of the vaccine antigen [47,48,49].

## 4. Conclusions

In summary, this study intended to call attention to some of the issues that hinder effective and efficient FMD control in Egypt and elsewhere in North Africa. As we described, there is insufficient information about circulating viruses as well as about the available and used vaccine strains, there may be no appropriate vaccine against the circulating serotype A viruses and there are concerns over biosafety in domestic vaccine production.

## Figures and Tables

**Table 1:** New sequence data from this study.

ID	Serotype	Sample Material	Origin	Year	Host	Sequence	Accession Number
						Type	
sample 8	A	saliva	Dakahlia	2016	cow	1D/VP1	MT863264
sample 9	A	saliva	Damietta	2016	cow	1D/VP1	MT863265
sample 10	A	saliva	Damietta	2016	buffalo	1D/VP1	MT863266
		culture supernatant	-	-	-	whole genome	MT863268
sample 11	A	saliva	Dakahlia	2016	buffalo	1D/VP1	MT863267
FTA card	O	inactivated vaccine	Egypt	2019		whole genome	MT863269

**Table 2:** Pairwise nucleotide identity between the 2016 serotype A isolate from this study (sample 10) and A/El-Fayoum/Egypt/2014 (accession# KP940474) or O/AMU_200/Uganda/2017 (accession# MH791293), respectively. Apart from the comparison across the entire ORF, each coding region was compared separately. For each region, the more similar strain is highlighted in gray.

Isolate	ORF	L	1A	1B	1C	1D	2A	2B	2C	3A	3B_1_	3B_2_	3B_3_	3C	3D
A/El-F/2014	87.3	88.1	89.8	89.3	90.5	86.6	98.2	93.9	83.9	78.2	76.8	79.2	83.3	87.2	88.3
O/AMU_200	85.9	76.3	85.1	72.5	73.9	64.9	94.4	94.4	94.9	91.9	98.6	87.5	94.4	95.3	94.9

## Supplementary Materials

The following are available online at https://www.mdpi.com/1999-4915/12/9/990/s1, Figure S1: Phylogenetic tree of full-ORF sequences of serotypes O, A and SAT2; Figure S2: Phylogenetic tree of leader-coding sequences of serotypes O, A and SAT2; Figure S3: Phylogenetic tree of P1-coding sequences of serotypes O, A and SAT2; Figure S4: Phylogenetic tree of 2A-coding sequences of serotypes O, A and SAT2; Figure S5: Phylogenetic tree of 2B-coding sequences of serotypes O, A and SAT2; Figure S6: Phylogenetic tree of sequences coding for non-structural proteins 2C to 3D of serotypes O, A and SAT2.
