# Supplementary figures and images for: Sequence Analysis of Egyptian Foot-and-Mouth Disease Virus Field and Vaccine Strains: Intertypic Recombination and Evidence for Accidental Release of Virulent Virus
Source: Viruses. 2020 Sep 6;12(9):990. doi: 10.3390/v12090990 (PMC7552000; doi:10.3390/v12090990)

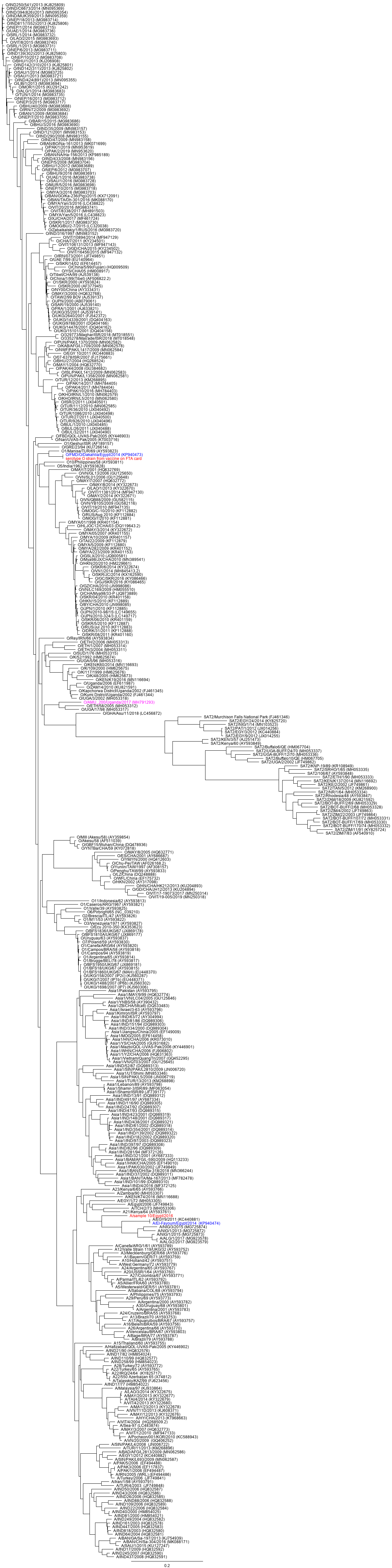

Supplement: Supplementary file 1 [file viruses-12-00990-s001.zip › Fig S1_full ORF tree.png]

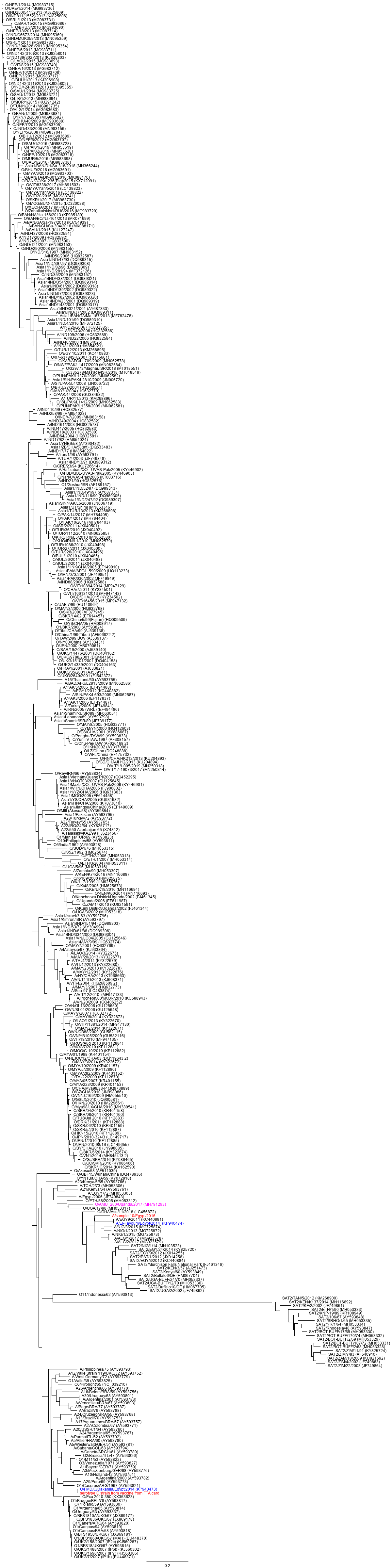

Supplement: Supplementary file 1 [file viruses-12-00990-s001.zip › Fig S2_Leader tree.png]

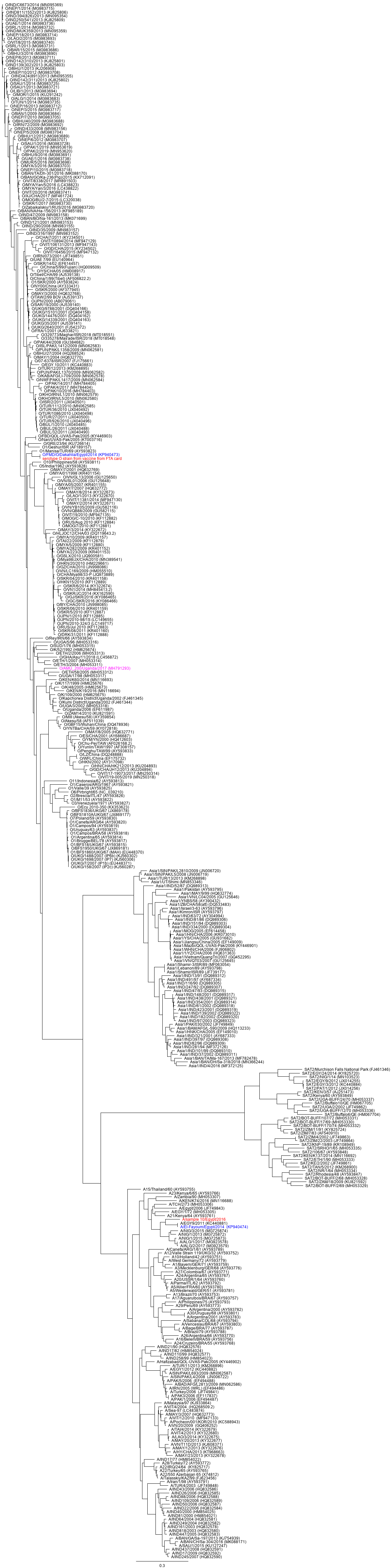

Supplement: Supplementary file 1 [file viruses-12-00990-s001.zip › Fig S3_P1 tree.png]

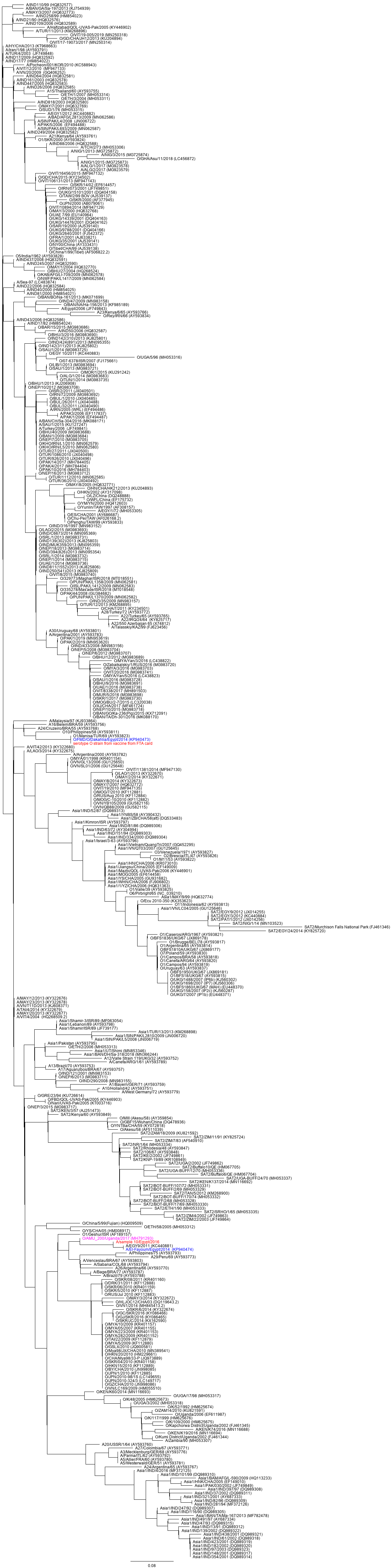

Supplement: Supplementary file 1 [file viruses-12-00990-s001.zip › Fig S4_2A tree.png]

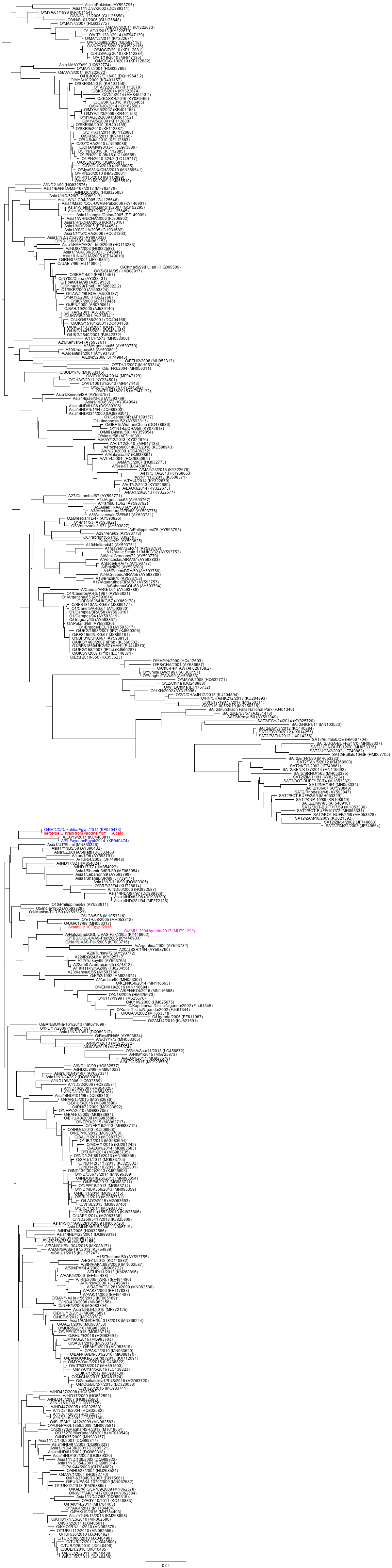

Supplement: Supplementary file 1 [file viruses-12-00990-s001.zip › Fig S5_2B tree.png]

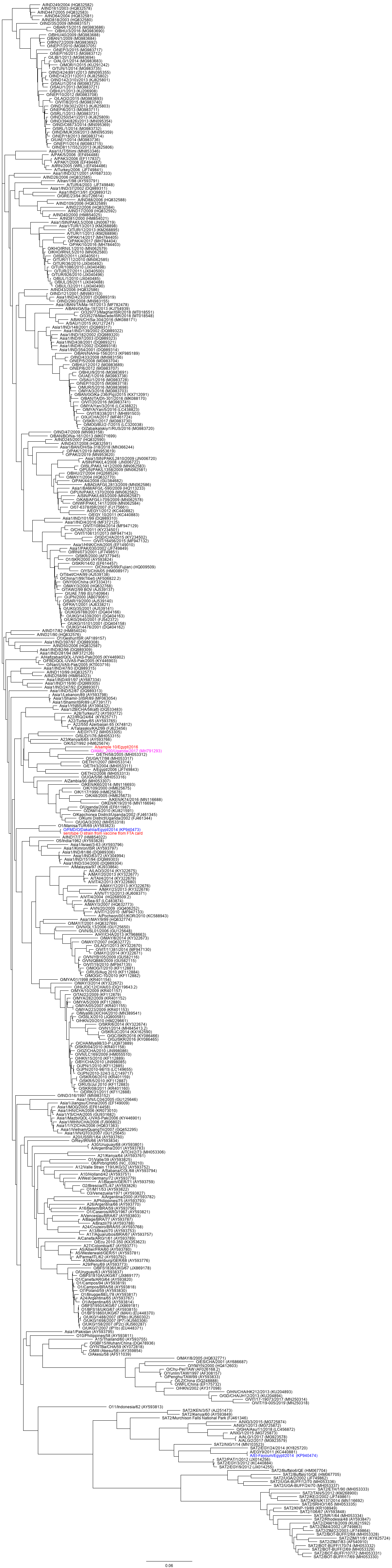

Supplement: Supplementary file 1 [file viruses-12-00990-s001.zip › Fig S6_2C3D tree.png]
